# Six Decades of History of Hypertension Research at the University of Toledo: Highlighting Pioneering Contributions in Biochemistry, Genetics, and Host-Microbiota Interactions

**DOI:** 10.1007/s11906-022-01226-0

**Published:** 2022-10-27

**Authors:** Veda Gokula, David Terrero, Bina Joe

**Affiliations:** 1 Center for Hypertension and Precision Medicine, Department of Physiology and Pharmacology, College of Medicine and Life Sciences, University of Toledo College of Medicine and Life Sciences, Block Health Science Building, 3000 Arlington Ave, Toledo, OH 43614-2598 USA; 2 Department of Pharmacology and Experimental Therapeutics, College of Pharmacy, University of Toledo, Toledo, OH USA

**Keywords:** Blood pressure, Cardiovascular, Renal, Microbiota, Genetics, Epigenetics

## Abstract

**Purpose of Review:**

The study aims to capture the history and lineage of hypertension researchers from the University of Toledo in Ohio and showcase their collective scientific contributions dating from their initial discoveries of the physiology of adrenal and renal systems and genetics regulating blood pressure (BP) to its more contemporary contributions including microbiota and metabolomic links to BP regulation.

**Recent Findings:**

The University of Toledo College of Medicine and Life Sciences (UTCOMLS), previously known as the Medical College of Ohio, has contributed significantly to our understanding of the etiology of hypertension. Two of the scientists, Patrick Mulrow and John Rapp from UTCOMLS, have been recognized with the highest honor, the Excellence in Hypertension award from the American Heart Association for their pioneering work on the physiology and genetics of hypertension, respectively. More recently, Bina Joe has continued their legacy in the basic sciences by uncovering previously unknown novel links between microbiota and metabolites to the etiology of hypertension, work that has been recognized by the American Heart Association with multiple awards. On the clinical research front, Christopher Cooper and colleagues lead the CORAL trials and contributed importantly to the investigations on renal artery stenosis treatment paradigms. Hypertension research at this institution has not only provided these pioneering insights, but also grown careers of scientists as leaders in academia as University Presidents and Deans of Medical Schools. Through the last decade, the university has expanded its commitment to Hypertension research as evident through the development of the Center for Hypertension and Precision Medicine led by Bina Joe as its founding Director.

**Summary:**

Hypertension being the top risk factor for cardiovascular diseases, which is the leading cause of human mortality, is an important area of research in multiple international universities. The UTCOMLS is one such university which, for the last 6 decades, has made significant contributions to our current understanding of hypertension. This review is a synthesis of this rich history. Additionally, it also serves as a collection of audio archives by more recent faculty who are also prominent leaders in the field of hypertension research, including John Rapp, Bina Joe, and Christopher Cooper, which are cataloged at Interviews.

## Founding and Biomedical Research at the Medical College of Ohio (MCO)

**Fig. 1:**
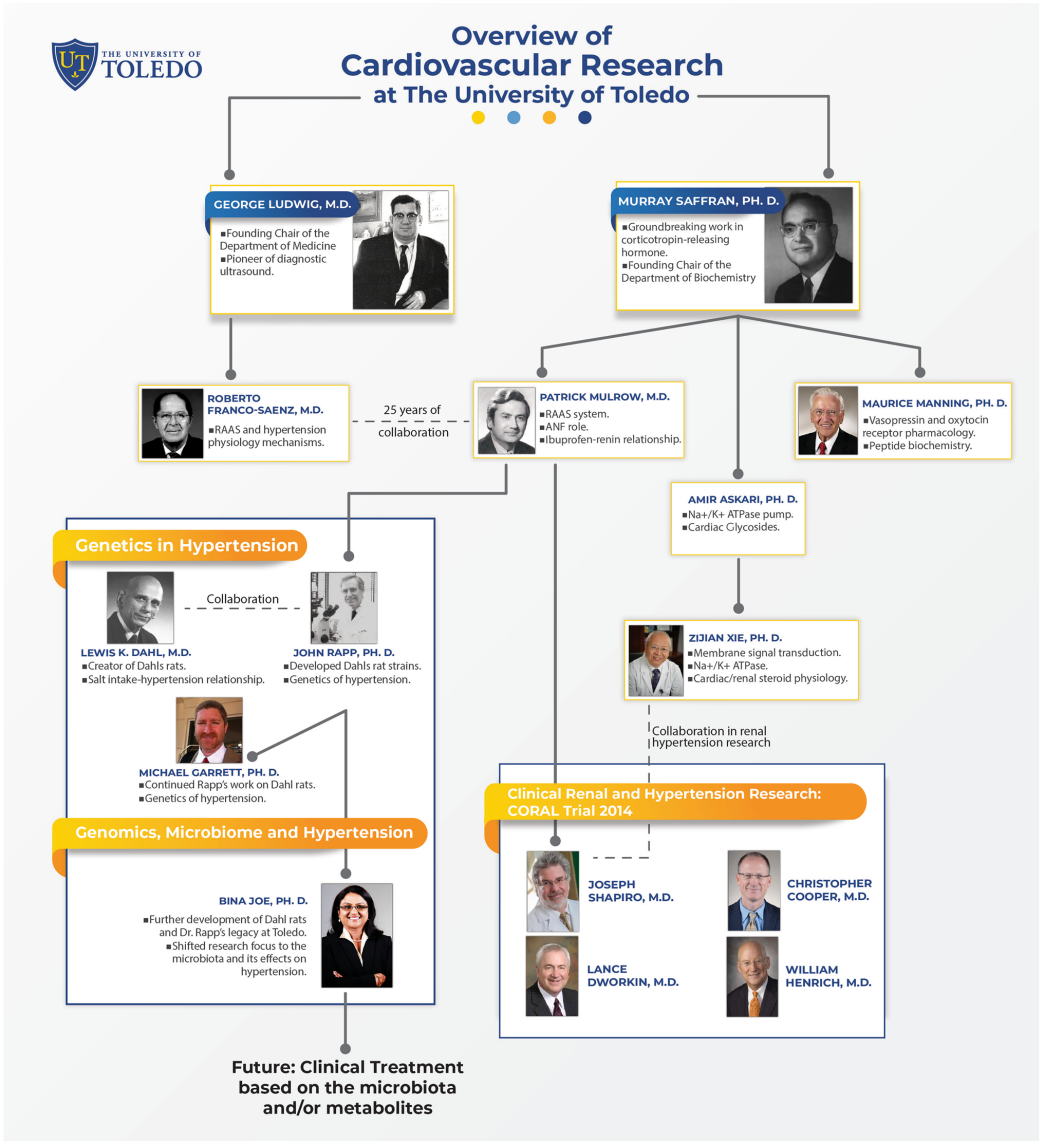
Flowchart of the history of cardiovascular research at The University of Toledo. Initial discoveries in endocrinology and nephrology over the last six decades have paved the way for future research in genomics, microbiome, and hypertension

**Fig. 2:**
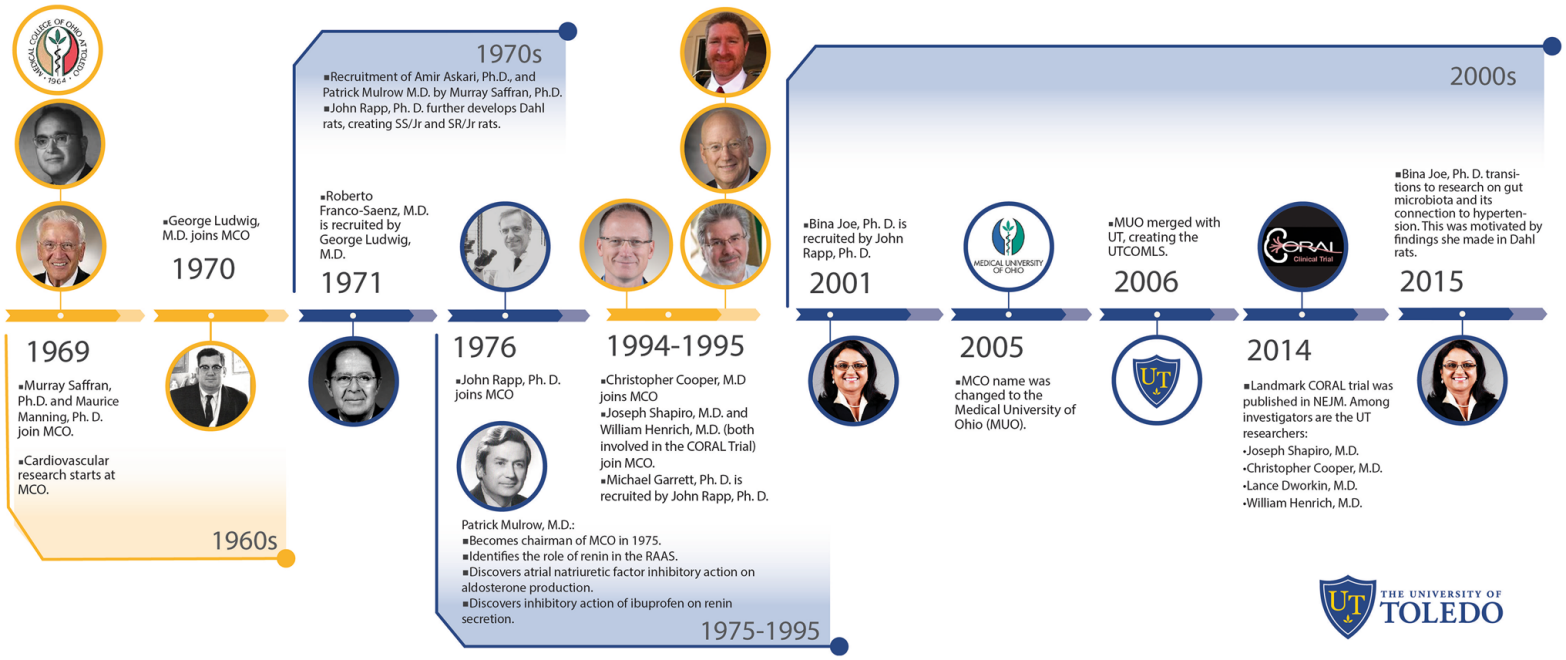
Timeline of hypertension research at The University of Toledo (UT), formerly known as the Medical College of Ohio (MCO). There is a long-standing history of hypertension research at UT that began in the 1970s and continues to grow today

The University of Toledo College of Medicine and Life Sciences (UTCOMLS) (https://www.utoledo.edu/med/) was originally known as the Medical College of Ohio (MCO). MCO was founded in 1964 but its first class of medical students began their studies in 1969 [1]. MCO has a history of innovative researchers in the cardiovascular field since its founding in 1964 (Figs. 1, 2, and Table 1). MCO was Northwest Ohio’s first independent medical school since the closure of the Toledo Medical College (1882–1914) (https://www.utoledo.edu/library/canaday/findingaids1/UM_68.pdf).

The foundations of basic cardiovascular research at MCO were laid by Murray Saffran, Ph.D., a renowned neuroendocrinologist [2–6] who arrived in 1969 and was the founder of the Biochemistry Department. Before arriving at MCO, Saffran worked at McGill University, Montreal, Canada, where he helped uncover the role of cortisol as a regulator of the body’s response to stress [2–6]. At MCO, Saffran continued his endocrinology research and explored the role of insulin in the vascular complications of diabetes [7–11]. Throughout the 1970s, he laid the foundation for a world-class cardiovascular and biochemical research department.

Also in 1970, George D. Ludwig, M.D., from the University of Pennsylvania joined MCO in 1970 as the founding chairman of the Department of Medicine. Although Ludwig’s expertise was on metabolic, endocrine, and molecular diseases with contributions in the areas of the abnormal heme, porphyrin, indoles, calcium-phosphate metabolism, parathyroid diseases, and inborn errors of metabolism, he is most remembered as a pioneer of the clinical application of the ultrasound technology. Tragically, Ludwig died of a cerebral hemorrhage in 1973, bringing a brilliant career to an untimely end at the age of 51 (https://www.ob-ultrasound.net/ludwig.html).

Meanwhile, Murray Saffran recruited Maurice Manning, Ph.D., from McGill University; Amir Askari, Ph.D., from Cornell University; and Patrick Mulrow, Ph.D., from Yale University (Fig. 1). Mulrow played a critical role in commencing hypertension research at MCO.

Manning began working in MCO in 1969 and was a faculty member for MCO’s inaugural class. He has made seminal contributions to advance the pharmacology of oxytocin and vasopressin [12–40]. During the course of his research, he has donated thousands of samples of oxytocin and vasopressor analogs from his laboratory to other researchers across the world and is internationally known for his expertise in peptide biochemistry. To-date, Manning remains as an Emeritus faculty member of UTCOMLS.

## Na^+^/K^+^ ATPase Pump Physiology

Amir Askari, Ph.D., pursued pioneering research related to cardiac glycoside-sensitive proteins and their effect on the cardiac Na^+^/K^+^ ATPase pump [41–43]. Askari investigated the pharmacologic properties of digitalis and how the drug affected the activity of the Na^+^/K^+^ ATPase pump. In 1990, Zijian Xie arrived at Toledo as a post-doctoral fellow in Askari’s laboratory. Xie continued working in MCO and further advanced research in Na^+^/K^+^ ATPase and cell membrane signal transduction [43–48, 49•]. Though the physiologic function of the pump has been studied for decades, the signaling capabilities of the pump were identified as a new function independent of the pump activity to maintain intracellular sodium and potassium homeostasis [49•, 50]. The research at Toledo to investigate how the Na^+^/K^+^ ATPase pump affected neighboring membrane proteins and signaling cascades was a unique aspect of its role in cardiovascular health. Unfortunately, both Askari and Xie passed away in 2020.

## From Endocrinology to Cardiovascular Research

Patrick Mulrow, M.D. is a Cornell and Stanford trained endocrine physician well known for his research on the renin-angiotensin aldosterone system (RAAS). Mulrow’s research was instrumental in proving that angiotensin II stimulated aldosterone secretion, and not adrenocorticotrophic hormone [51–56]. Mulrow trained at the University of Pennsylvania and was recruited to Toledo in 1971 by Ludwig (Table 1). At Toledo, Mulrow conducted hypertension research in collaboration with Roberto Franco-Saenz, M.D., for 25 years [51–79]. Dr. Franco-Saenz was an enthusiastic physician and scientist. Many of his publications in Toledo were focused on the renin-angiotensin system and mechanisms of hypertension. In 1975, Mulrow became Chair of Medicine at MCO (Fig. 2). As Chair from 1975 to 1995, Mulrow continued his physiology research and expanded hypertension research in the department (Table 1). Mulrow and Franco-Saenz discovered that atrial natriuretic factor, a heart hormone, inhibited aldosterone production. Mulrow’s research along with his penchant for leadership in a variety of scientific organizations such as chairman of the American Heart Association’s (AHA) Council for High Blood Pressure Research, chairman of the Medical Research Council in Canada, president of the Central Society for Clinical Research, member of the US National Research Council, Member of the Board of Directors of the Inter‐American Society of Hypertension, and involvement with the National High Blood Pressure Education Program (NHBPEP) gained national and international visibility to MCO.

## Clinical Hypertension Research

Mulrow had a productive career at MCO in both cardiovascular and renal hypertension research. He found that NSAIDs like indomethacin or naproxen could inhibit renin secretion and lead to kidney damage in animals as well as patients with volume depletion [80, 81]. This was an early link to an NIH-funded *National Analgesic Nephropathy Study,* which would later be led by the nephrologists Joseph Shapiro, M.D., who came to MCO in 1995 and William Henrich, M.D., who worked at MCO from 1995 to 1999 [82–90]. Henrich is now President of the University of Texas Health Science Center, San Antonio, TX.

In 1999, Shapiro was appointed the Chair of Medicine at MCO. He continued to foster the research environment that Mulrow had established over the last 3 decades and researched on various topics pertinent to hypertension [91, 92, 93••, 94–119]. Shapiro served as Chair until 2012, and subsequently moved as the Dean at Marshall University College of Medicine.

Between 2004 and 2008, MCO went through two name changes from the Medical University of Ohio to what is now referred to as the University of Toledo College of Medicine and Life Sciences. Therefore, references beyond these years are likely to not refer to MCO anymore.

Renal research at UTCOMLS had a high point in 2014 with the publication of The Cardiovascular Outcomes in Renal Atherosclerotic Lesions (CORAL) trial [93••]. The CORAL trial, led by Christopher Cooper, Lance Dworkin, William Henrich, and Joseph Shapiro, evaluated the clinical outcomes of renal artery stents in patients with renal artery stenosis. Renal artery stenosis is present in 1–5% of patients with hypertension and can occur in the setting of other cardiovascular diseases and comorbidities. Results of the trial indicated that renal artery stents did not significantly decrease the incidence of adverse cardiovascular events [93••]. This study was published in the New England Journal of Medicine in 2014 and was one of the top 5 cardiovascular studies in that year [93••]. Results of the CORAL trial provided important data regarding the clinical management of these patients and were incorporated into the 2017 American College of Cardiology (ACC)/AHA Hypertension Guidelines [120] for the treatment of hypertensive patients. Cooper, who is now the Dean of the College of Medicine and Life Sciences stated, “I think I’ve done a tiny little piece of unraveling the biology of people. I think the better we understand the biology of people, the better we can take care of the people we serve.”

## Learning the Genetics of Hypertension

In addition to the physiological and clinical studies, this institution is internationally reputed for its pioneering contributions to the dissection of genetic elements causing hypertension in experimental rats [121, 122••]. This new area of research was initiated by John Rapp, who was recruited to MCO by Mulrow. Rapp had acquired unique rat strains called the Dahl rats [123]. The Dahl rats are named after Lewis Kitchener Dahl, M.D., a physiologist who discovered the association between salt intake and hypertension. Dahl selectively bred the rats for salt sensitivity (S) and salt resistance (R), with the goal being to determine if there were genetic differences between these two strains of rats [124–130]. For details on Dahl and the history of these strains, readers are referred to the 2014 Dahl lecture award article [123]. It is important to note that their findings preceded the now common knowledge that a high-salt diet is associated with hypertension. In 1976, Mulrow recruited Rapp, who brought his Dahl rats to MCO. MCO was a new medical college at the time. The major focus of Rapp’s research at Toledo was uncovering the causal genes for the pathogenesis of salt-sensitive hypertension. Throughout the 1970s, he was primarily focused on the inbreeding of the S and R rat strains. This helped maintain genetic uniformity and the stability of the traits in each strain.

Rapp laid the groundwork for understanding their genetics by inbreeding these rats to develop the Dahl salt-sensitive (S) and Dahl salt-resistant (R) rats [131]. These are to-date among the most widely used inbred models for studies on hypertension as they are the only rat models with direct clinical relevance to salt-sensitive hypertension. The original colonies of the Dahl S and R strains are maintained in-house at the University of Toledo College of Medicine and Life Sciences. The inbred strains are now officially designated as SS/Jr and SR/Jr for salt-sensitive and salt-resistant, and Jr stands for John Rapp. These rats have been recently licensed to Charles River Laboratories and registered at the rat genome database as SS/Jr/Tol rats (https://rgd.mcw.edu/rgdweb/report/strain/main.html?id=724573).

Rapp hypothesized that a few major genes would be involved in the pathogenesis of hypertension and was focused on the reproducibility of his results. He performed breeding experiments to determine which chromosomes and what portions of the chromosomes were involved in the genetic predisposition to salt-sensitive hypertension. It was important to identify segments of chromosomes where a gene might be located. This research occurred before the human genome project, and consequently, widespread sequencing was not available.

Using these rats, Rapp created maps of chromosomes to find the areas of the chromosomes that were segregated by blood pressure, known as quantitative trait loci (QTLs). His outstanding work on discovering multiple such QTLs [121] was lauded by the AHA Council for High Blood Pressure Research, which decorated him with its highest honor, the Excellence in Hypertension Research Award (which was then referred to as the Novartis award). He became Chair of the Physiology department in 1994 and helped reshape the focus of the department to cardiovascular genetics. Dr. Rapp had two trainees that would go on to make significant contributions to the field of hypertension in their own right: Bina Joe and Michael Garrett.

In 1995, Rapp recruited Garrett to Toledo as a research assistant for his laboratory, who worked with Rapp to advance the genetic analysis of renal disease in the Dahl S rat. Joe was recruited by Rapp as Research Faculty in 2001. Prior to coming to Toledo, Joe, who graduated from Mysore University in India, was a Fogarty fellow at the Intramural division of the NIH. While at the NIH, she was conducting research on the genetics of rheumatoid arthritis using experimental congenic rat models. After many years of collaboration in Toledo, Rapp, Garrett, and Joe identified multiple genetic loci that causally regulated blood pressure in the Dahl S rat [121, 132–145]. They continued to work together until Rapp retired in 2004. In 2007, Garrett left Toledo, moved to the Medical College of Wisconsin, and moved again to the University of Mississippi Medical Center, where he is a Professor. He continues to work on the Dahl S rats to delineate the genetics of kidney disease [146–166]. The Joe lab in Toledo further advanced this positional cloning research by combining it with gene-editing technology to identify many protein coding genes and non-coding genes as BP QTLs [132–145].

In tracing this “genealogy,” it is important to mention that there are multiple varieties of the Dahl S rats which are genetically distinct and present with varying extents of hypertension [167]. Researchers who intend to use the original stock of Dahl S rats, which were inbred by Rapp, may please note that these are currently available through two academic sources, the Joe laboratory at the University of Toledo (https://www.utoledo.edu/med/depts/physpharm/faculty/joe.html) and the Garrett laboratory at the University of Mississippi Medical Center (https://www.umc.edu/som/Departments%20and%20Offices/SOM%20Departments/Pharmacology%20and%20Toxicology/Faculty/Michael-Garrett.html). Besides these 2 sources, a commercial source is the Charles River Laboratory as the Dahl S rat was recently licensed to the Charles River Laboratory by Rapp, which was facilitated by Joe.

## From Genetics to the Gut Microbiota in Hypertension

Close to a decade after the human and rat genome sequences were decoded, the Joe lab was successful in positional cloning of genetic loci regulating BP in the Dahl rat [122••, 135, 136, 168, 169]. However, genome-wide association studies in humans as well as QTL mapping studies in rats shed light on the landscape of the genetics of hypertension to be much larger than what was originally anticipated. More than 1500 loci in humans [170] and > 500 loci (https://rgd.mcw.edu/) [122••] in rats were located as potential regions of the mammalian genome to harbor BP regulatory genes. As such, contemplating clinical targets to render genetic corrections was not feasible. The value of identifying the polymorphisms was therefore pivoted by human geneticists towards the development of a polygenic risk score for predictive individualized medicine. Meanwhile, intrigued by a Science publication in 2010 that mice lacking Tlr5 [171], a bacterial flagellin receptor, developed microbial dysbiosis and metabolic syndrome, the Joe laboratory hypothesized that beyond the genetics of hypertension, microbiota, which are sensitive to salt and antibiotics, is a factor contributing to BP regulation. The hypothesis was indeed proven correct by the demonstration via cecal transplantation studies that the Dahl S rats increased BP when gavaged with cecal microbiota from the Dahl salt-resistant (R) rat [172••, 173]. Since the publication of this pioneering study in 2015, the University of Toledo is currently regarded as the site of discovery of the important link between gut microbiota and hypertension [172••, 174–188]. More recently, Joe et al. have employed germ-free rats and reported that gut microbiota is obligatory to blood pressure homeostasis [179].

Yet another significant contribution from the Joe laboratory is the discovery of the strong link between metabolism [189•], especially the inverse relationship between the ketone body, betahydroxybutyrate (BHB), and hypertension [190]. Both renal and vascular mechanisms have been identified via BHB facilitating the inhibition of the Nlrp3 inflammasome and vasodilatory function, respectively [191–193].

Overall, these impactful contributions were recognized by the Hypertension research community in the form of the Lewis Dahl Lectureship award in 2014 [123] and the Harriet Dustan Award in 2019 [189•] to Joe. It is notable that in both categories, Joe is globally, the first woman of color awardee of both of these awards.

## Current Hypertension Research

Building on this strong foundation of both basic and clinical sciences, in 2011, the University-wide Research Council approved the Center for Hypertension and Precision Medicine. Most importantly, in 2015, fueled by a significant 50-year legacy model affiliation with a local Health organization, ProMedica (https://www.promedica.org/service-to-the-community/ut-academic-affiliation), the Dean of the College of Medicine, Cooper, and the Director of Hypertension Research, Joe, both Distinguished University Professors of the University, have expanded talent acquisition for hypertension research. Notable recruits include Matam Vijay-Kumar, who initially discovered the link between Tlr5 receptors and blood pressure [171, 172••, 173–180, 183–185, 187, 188, 189•, 191, 192, 194–205], [181, 196]; Jasenka Zubcevic [206–244], who focuses on the gut-brain axis; Jennifer Hill, whose work is on prenatal environmental effects on hypertension [182, 186, 189•, 196]; Tao Yang, who is studying microbiota as causes for drug resistance in hypertensives [175, 179, 181, 187, 189•, 192, 209, 210, 223, 229–234, 239]; Charles Thodeti and Guillermo Vazquez, who focus on transient receptor potential channels [245–283]; Islam Osman, who is a vascular physiologist [284–296]; Lauren Koch, who has developed unique rat models with distinct aerobic exercise endurance capacities: low- and high-capacity runners (LCR and HCR) to study the relationship between exercise and hypertension [188, 189•, 199]; Sailaja Paruchuri, who works on lipid mediators [245, 246, 248–252, 255, 257, 258, 261, 263–265]; and Piu Saha, who is working in immunological aspects promoting hypertension in rat genetic models [174, 175, 179, 180, 187, 191, 194, 195, 197, 200, 201, 205]. From the Department of Medicine, Cooper and his colleagues including Lance Dworkin, David Kennedy, Steven Haller, and Rujun Gong continue their investigations on the renal physiology of BP control [101, 102, 104, 112, 297–339]. From the College of Pharmacy, members of the Center for Hypertension and Precision Medicine include Wissam Aboualaiwi [340–350] in the Department of Pharmacology and Experimental Therapeutics who investigates drug targets and the physiology of cilia in polycystic kidney disease and hypertension. Further, in 2018, UTCOMLS identified Hypertension as a strategically focused spotlight area of unique distinction.

**Table 1:**
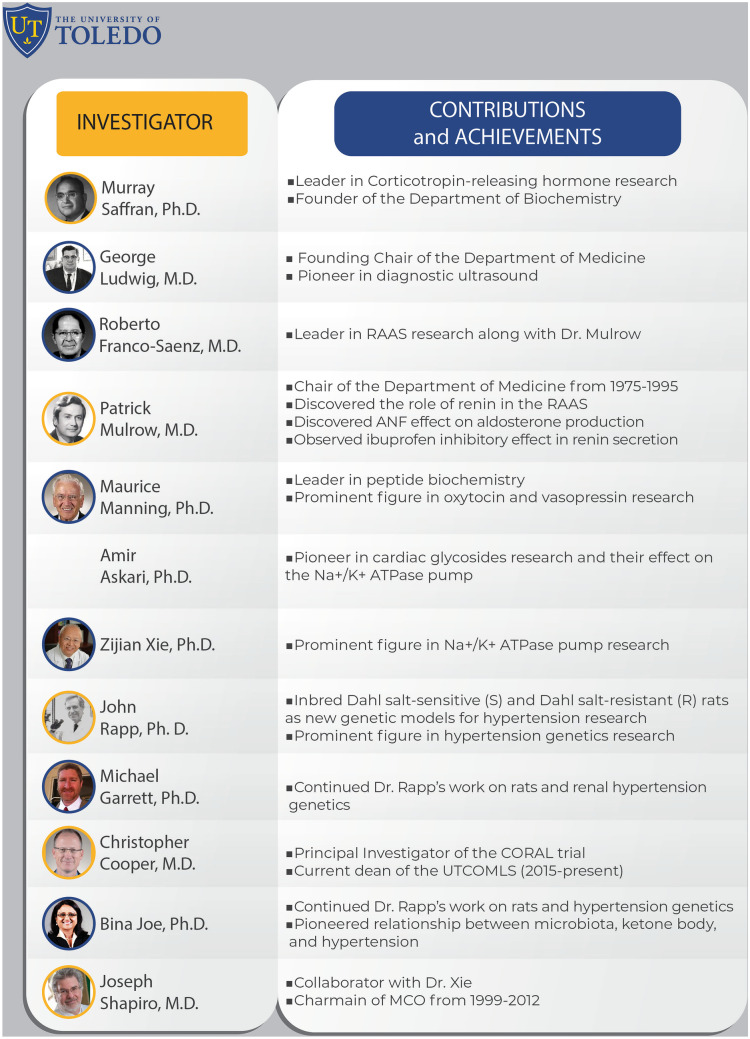
Highlights of contributions and achievements of investigators at UT
